# Bifunctional Chromium-Doped Phenolic Porous Hydrothermal Carbon Catalysts for the Catalytic Conversion of Glucose to 5-Hydroxymethylfurfural

**DOI:** 10.3390/ijms26083648

**Published:** 2025-04-12

**Authors:** Pize Xiao, Wei Mao, Zhiming Wu, Huimin Gao, Chutong Ling, Jinghong Zhou

**Affiliations:** Guangxi Key Laboratory of Clean Pulp & Papermaking and Pollution Control, School of Light Industrial and Food Engineering, Guangxi University, Nanning 530004, China; 2216301047@st.gxu.edu.cn (P.X.); maowei@st.gxu.edu.cn (W.M.); 2216391048@st.gxu.edu.cn (Z.W.); 2216391010@st.gxu.edu.cn (H.G.); lingchuttt@st.gxu.edu.cn (C.L.)

**Keywords:** 5-hydroxymethylfurfural, phenolic-resin-based carbon, catalysis, glucose

## Abstract

A sustainable and efficient approach for converting carbohydrates into 5-hydroxymethylfurfural (HMF) via heterogeneous catalysis is crucial for effectively utilizing biomass. In this study, we synthesized a series of CrX-polyphenol-formaldehyde resin (PTF) catalysts, which are composites of Cr-doped phenolic-resin-based hydrothermal carbon, using a chelation-assisted multicomponent co-assembly strategy. The performance of the synthesized catalysts was assessed through various analytical techniques, including scanning electron microscopy, transmission electron microscopy, thermogravimetric analysis, X-ray photoelectron spectroscopy, pyrolysis–Fourier transform infrared spectroscopy, and Brunauer–Emmett–Teller analysis. Cr incorporation into the catalysts enhanced the total and Lewis acidities. Notably, the optimized catalyst, designated as Cr0.6-PTF, achieved an effective glucose conversion into HMF, yielding a maximum of 69.5% at 180 °C for 180 min in a saturated NaCl solution (NaClaq)/dimethyl sulfoxide (2: 18) solvent system. Furthermore, Cr0.6-PTF maintained excellent catalytic activity and a stable chemical structure after nine cyclic reactions, resulting in a 63.8% HMF yield from glucose. This study revealed an innovative approach for utilizing metal-doped phenolic resin hydrothermal carbon to transform glucose into platform chemicals.

## 1. Introduction

With the rapid industrial development, growing demand for energy, and progressive depletion of non-renewable fossil fuels, it is imperative to identify sustainable alternatives to mitigate energy and resource scarcity [1,2]. Biomass, as a renewable resource, can potentially be transformed into various chemicals and fuels [3]. Bio-refining, which utilizes biomass-derived sugars to produce liquid biofuels and chemicals, is a promising strategy for ensuring energy security and environmental sustainability [4,5]. 5-Hydroxymethylfurfural (HMF) functions as an intermediate in the production of fine chemicals and within the biorefinery process [6]. The United States Department of Energy has recognized HMF as a pivotal biomass-derived platform chemical due to its unsaturated structure comprising a furan ring, hydroxyl group, and aldehyde moiety [7,8,9,10].

HMF is synthesized via fructose and glucose dehydration. Although high HMF yields have been achieved from fructose, its commercialization is not economically viable because of its high cost and scarcity. Consequently, glucose is a more desirable source for HMF synthesis because it is a more accessible, cheaper, and more abundant feedstock than fructose [11]. Glucose undergoes a two-step reaction, initially isomerized to fructose through Lewis acid catalysis, followed by dehydration to form HMF, mediated by Brønsted acid [12]. Homogeneous catalysts, such as Cr^3+^, Fe^3+^, Al^3+^, and Sn^4+^ halides [13], have been utilized to transform sugars into HMF [14,15,16,17]. Zhou et al. [16] studied the effect of various catalysts, including FeCl_3_·6H_2_O, CrCl_3_·6H_2_O, and AlCl_3_, combined with different solvents. The results revealed that the highest HMF yield (54.4%) was achieved using CrCl_3_·6H_2_O in dimethyl sulfoxide (DMSO) [16]. Zhao et al. [18] found that CrCl_3_ effectively catalyzed glucose dehydration, achieving a 70% HMF yield in a chlorinated 1-alkyl-3-methylimidazole solution [18]. However, homogeneous catalytic systems have challenges, such as product separation, catalyst recovery, and reactor corrosion. Contrastingly, heterogeneous catalysts are easy to separate and reuse and are desirable for converting biomass into HMF, particularly for glucose-to-HMF reactions [19,20,21,22,23].

Previous studies have demonstrated that biochar is an excellent heterogeneous catalyst or catalyst support material due to its rich functional groups, large specific surface area, and adjustable pore structure [24,25]. Additionally, metal catalysts supported by hydrochars have demonstrated high catalytic activity for glucose isomerization [26,27]. Tannic acid (TA), which is rich in catechol and gallic acid, behaves similarly to phenol or resorcinol, readily reacting with aldehydes to form polyphenol-formaldehyde resins. These resins are widely used as precursors for synthesizing porous carbon materials [28,29]. The catechol group in TA also exhibits strong chelation with metal species, making it suitable for preparing metal/metal oxide nanoparticle-decorated carbon materials. Metal-TA coordination polymers, a subclass of metal/phenolic resin complex, have recently gained widespread attention as multifunctional platforms [30]. Liu et al. [31] synthesized TA-formaldehyde (TAF) resin spheres utilizing TA and formaldehyde as reactants via the Stöber method. The abundant hydroxyl groups in TAF enable it to chelate metal ions, resulting in metal-modified carbon spheres after carbonization [31]. Wang et al. [32] developed a wet chemical sol-gel methodology to synthesize metal/TAF resin spheres. Through thermal decomposition, they produced mesoporous carbon spheres with uniformly distributed metal species that were capable of degrading organic pollutants [32]. Wei et al. also reported on the sol-gel synthesis of metal polyphenol formaldehyde resin and their derived porous metal–carbon spheres, which demonstrated high catalytic activity for oxygen reduction reactions. However, there are few studies on metal-doped polyphenol-formaldehyde-resin-based carbon material as catalysts for the conversion of glucose into HMF [30].

**Scheme 1:**
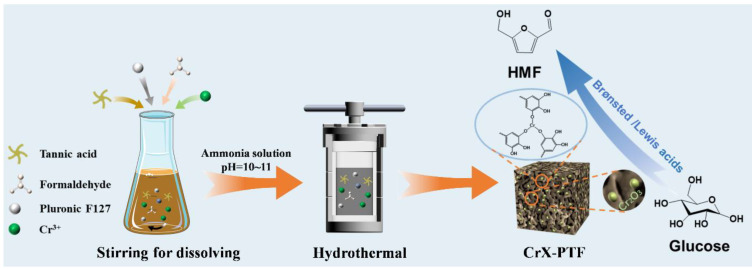
Synthetic of a Cr-doped phenolic resin porous hydrothermal carbon catalyst for HMF production.

In this study, TA was incorporated into the one-pot hydrothermal synthesis of polyphenol-formaldehyde resin (PTF) to introduce a high density of catechol groups capable of chelating Cr^3+^, resulting in the formation of a Cr-doped phenolic-resin-based porous carbon catalyst, designated as CrX-PTF. A schematic representation of this reaction is illustrated in Scheme 1. The resultant CrX-PTF composites demonstrated a markedly enhanced catalytic activity for converting glucose to HMF, which was attributed to their high Lewis acidity and total acid content. Moreover, the CrX-PTF catalyst demonstrated excellent reusability, with only a 6.5% decline in HMF yield after nine consecutive cycles. This exceptional stability is due to the strong anchoring effect between chromium nanoparticles (NPs) and the resin-based porous carbon matrix, highlighting its potential for industrial applications.

## 2. Results and Discussion

**Figure 1:**
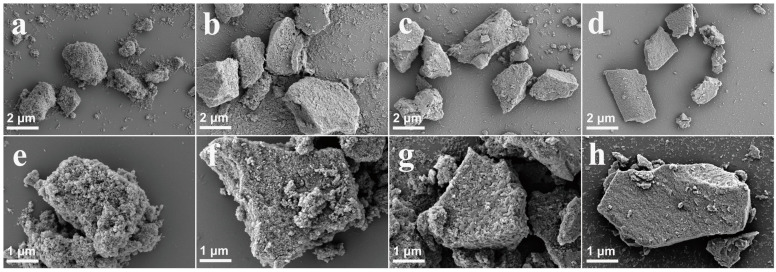
SEM images of the catalyst (**a**,**e**) Cr0.2-PTF, (**b**,**f**) Cr0.4-PTF, (**c**,**g**) Cr0.6-PTF, and (**d**,**h**) Cr0.7-PTF.

**Figure 2:**
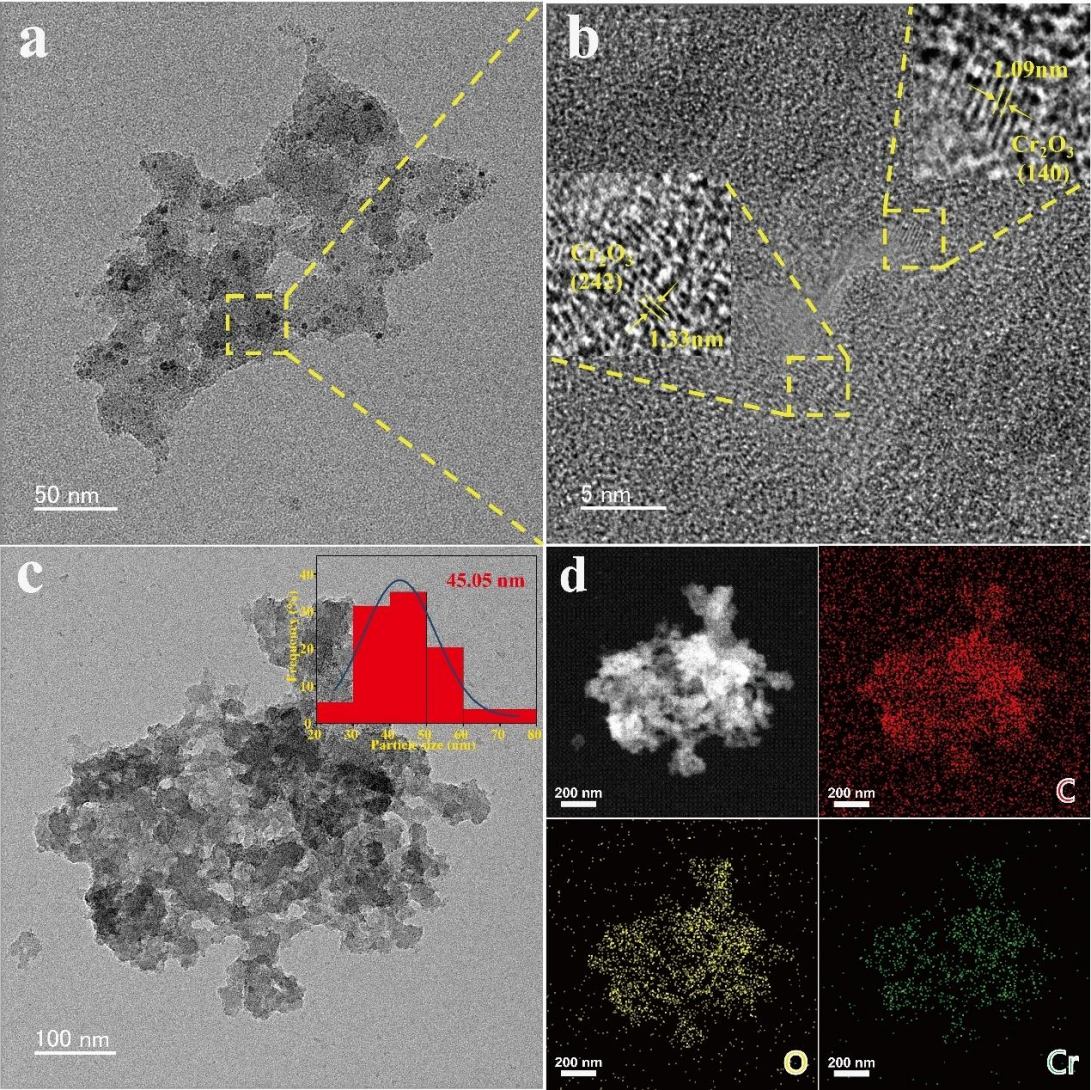
TEM images of Cr0.6-PTF (**a**–**c**). (**d**) EDX elemental mapping image of Cr0.6-PTF.

The micromorphological characteristics of the CrX-PTF surfaces were examined using SEM (Figure 1). SEM imaging revealed that Cr0.2-PTF and Cr0.4-PTF exhibited rough surfaces containing well-defined pore structures, while Cr0.6-PTF and Cr0.7-PTF displayed progressively smoother morphologies as Cr doping increased. This trend correlates with BET measurements, which show reduced surface area and pore volume at higher Cr ratios (Table 1). Cr0.6-PTF TEM images (Figure 2a–c) were obtained and complemented by the corresponding energy dispersive spectroscopy (EDS) mapping (Figure 2d) to provide insights into the internal structural characteristics of the catalyst. The Cr_2_O_3_ NPs were uniformly dispersed within the carbon matrix, with an average particle size distribution of approximately 45 nm (Figure 2a). Cr0.6-PTF exhibited numerous pores on the surface of its structured carbon (Figure 1), which was consistent with the BET results presented in Table 1. Further local magnification (Figure 2b) revealed that the Cr0.6-PTF lattice fringes were discerned in the TEM images, with calculated lattice spacings of 1.09 nm and 1.33 nm, corresponding to the (140) and (242) Cr_2_O_3_ crystal planes, respectively. The elemental distributions of carbon, oxygen, and Cr in the Cr0.6-PTF sample, as determined by EDS (Figure 2d), indicated the highly uniform distribution of Cr. The presence of oxygen (O) alongside chromium (Cr) strongly suggests the formation of chromium oxide species, which aligns with the crystallographic features observed in the TEM images.

The specific surface areas and pore-size distributions of the CrX-PTF catalysts are presented in Table 1 and Supplementary Figure S1. Significant variations in the specific surface area and pore size were observed among the catalysts with different Cr doping levels. The Cr0.2-PTF and Cr0.4-PTF catalysts exhibited type-IV isotherms, indicating their mesoporous nature [11]. Conversely, the Cr0.6-PTF and Cr0.7-PTF catalysts, prepared with higher Cr contents, exhibited mesoporous structures but reduced mesopores. The specific surface area of Cr0.2-PTF was 126.54 m^2^/g, with a mesopore volume of 0.57 cm^3^/g and a pore size of 18.09 nm (Table 1). In comparison, the PTF, Cr0.4-PTF, Cr0.6-PTF, and Cr0.7-PTF specific surface areas were 21.54, 97.31, 39.75, and 33.63 m^2^/g, respectively, with corresponding mesopore volumes of 0.12, 0.21, 0.06, and 0.05 cm^3^/g, and pore sizes of 17.61, 8.47, 6.14, and 4.11 nm, respectively, indicating that the addition of Cr ions could markedly increase the specific surface area of the catalyst and that an elevated concentration of Cr ions progressively reduced the specific surface area, mesopore volume, and pore size of the catalyst. This phenomenon resulted from the chelation of Cr by the hydroxyl groups of TA. As the Cr concentration increased, the complexation reaction intensified, blocking the mesopores, reducing the pore size, and increasing the density of agglomerates, all of which decreased the specific surface area of the catalyst. The distribution of pores and mesopores improved molecular diffusion, reduced mass transfer resistance, and enhanced substrate–active site interactions, thus accelerating product separation [33].

**Figure 3:**
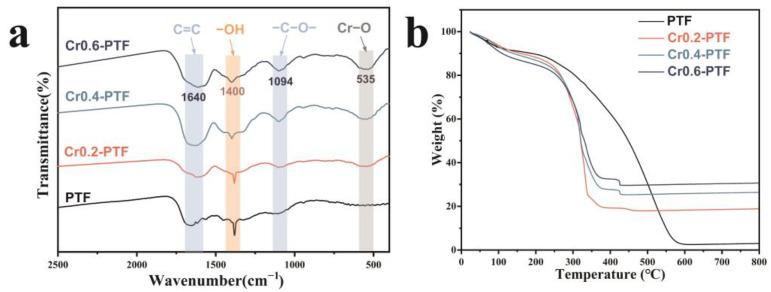
(**a**) FTIR spectra and (**b**) TGA plots of catalysts prepared with different Cr doping levels.

**Figure 4:**
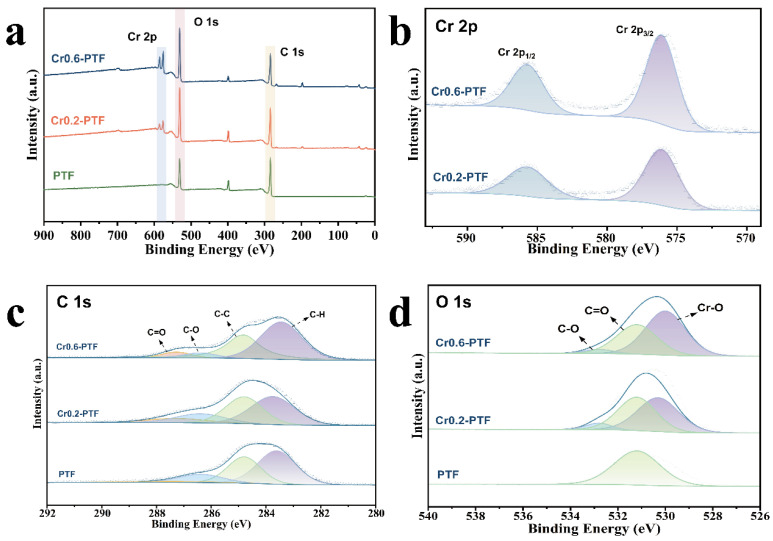
(**a**) XPS full, (**b**) Cr2p high-resolution, (**c**) C1s high-resolution, and (**d**) O1s high-resolution spectra of CrX-PTF (X = 0, 0.2, 0.6).

FTIR analysis was employed to elucidate the functional groups present in CrX-PTF. All samples exhibited characteristic peaks at 1640, 1400, and 1094 cm^−1^, which corresponded to the characteristic absorption peaks of the C=C, Ar-OH, and C-O- stretching vibrations of the benzene ring, respectively (Figure 3a) [20,34,35]. The peak at 535 cm^−1^ was the Cr-O characteristic absorption peak [36,37,38]. As the concentration of Cr increased, the intensity of the phenolic hydroxyl groups decreased, whereas that of the Cr-O characteristic peak increased. This phenomenon can be attributed to the chelation of Cr ions with the TA hydroxyl groups. The elemental compositions and chemical states of the catalysts were determined using XPS (Figure 4). The full-survey spectrum (Figure 4a) confirmed the presence of C and O in all samples. No Cr^3+^ characteristic peaks were found in the PTF sample; Cr was present only in CrCl_3_·6H_2_O-added samples (Cr0.2-PTF and Cr0.6-PTF). The high-resolution Cr 2p spectra (Figure 4b) further characterized the chemical state of Cr in these samples. A characteristic peak attributed to the Cr-O bond was observed at 530.2 eV [39]. Additionally, two characteristic peaks were observed at 576.1 and 585.7 eV, corresponding to Cr^3+^ (Cr2p3/2 and Cr2p1/2, respectively) [40]. The spin–orbit splitting value of these double peaks was 9.6 eV (BE = 585.7–576.1), which is consistent with that of Cr(III) [32,41,42]. The Cr-O peak area was 53.98% and 63.30% for Cr0.2-PTF and Cr0.6-PTF, respectively (Table S1). Combined with the FT-IR and TEM results, this indicates the formation of Cr_2_O_3_ in the Cr0.2-PTF and Cr0.6-PTF samples.

The thermal stabilities of the catalysts are presented in Figure 3b. All samples exhibited a minor weight loss below 100 °C, primarily due to the desorption of moisture and water molecules [43]. A significant weight loss of 90.38% was observed at 620 °C for PTF, with no further weight loss observed upon further temperature increases. The weight loss of the CrX-PTF sample was markedly lower than that of the PTF sample. At 440 °C, the weight losses were 81.12%, 73.59%, and 69.33% for Cr0.2-PTF, Cr0.4-PTF, and Cr0.6-PTF, respectively (Table S2). Further temperature increases resulted in no additional weight loss, indicating the complete breakdown of the catalyst and the presence of only metallic oxide. Furthermore, when the TA/CrCl_3_·6H_2_O mass ratio was 1:1, 1:2, 1:3, and 1:3.5, the Cr content in the residual catalyst, analyzed by ICP-OES, was 10.8%, 12.6%, 15.6%, 17.2%, and 18.2% (Table S5), respectively. These results confirm the effective incorporation of Cr into the catalyst.

**Figure 5:**
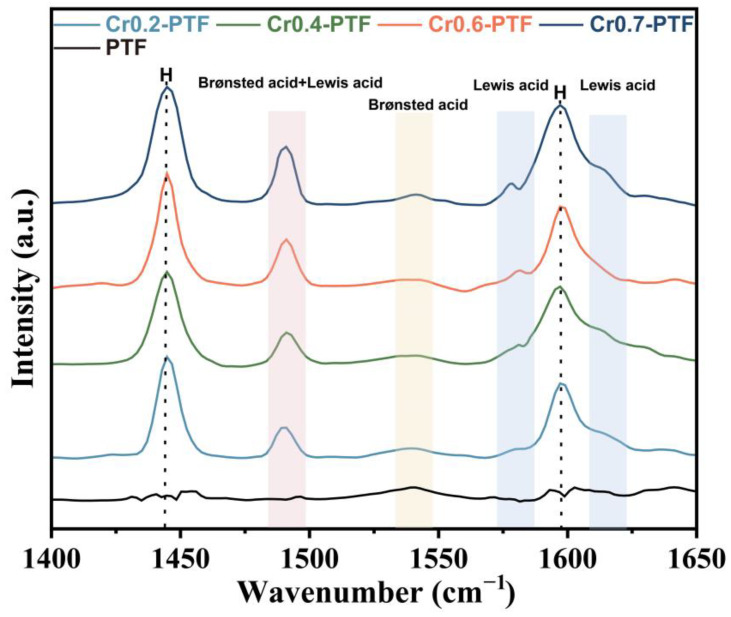
Py-FTIR spectra of CrX-PTF (X = 0.2, 0.4, 0.6, and 0.7) and PTF. Note: H represents hydrogen-bonded pyridine.

The types of acid sites and the amounts of acid in the prepared catalysts were determined using Py-FTIR (Figure 5). Cr introduction considerably enhanced the CrX-PTF Lewis and total acidities, with a marginal impact on Brønsted acidity (Table 2). Specifically, the amounts of Lewis acid in PTF, Cr0.2-PTF, Cr0.4-PTF, Cr0.6-PTF, and Cr0.7-PTF were 0, 34.73, 38.68, 46.62, and 54.43 μmol/g, respectively, indicating that Cr is a potent Lewis acid center. The intensity of the Lewis acids is positively correlated with glucose conversion because glucose isomerization is a reaction catalyzed by Lewis acids [44]. Glucose conversion progressively increased from 74% to 100% (Table S3)). Additionally, PTF had Brønsted acid sites only, whereas CrX-PTF had Brønsted and Lewis acid sites, probably because the Brønsted acidity originated from phenolic resin porous carbon, whereas the Lewis acidity stemmed from Cr_2_O_3_ [45]. TA provides additional protons through its phenolic hydroxyl groups, which chelate metal ions. Thus, the Brønsted acid sites originate from unreacted TA on the surface of the material [46]. Cr doping into phenolic resin porous carbon increases the number of Lewis acid sites, as Cr is recognized for its strong Lewis acid characteristics.

Solvents play a crucial role in heterogeneous catalytic reactions. To evaluate their impact on glucose conversion to HMF, various solvents were tested (Table S4), including dimethyl sulfoxide (DMSO), γ-valerolactone (GVL), N,N-dimethylformamide (DMF), N,N-dimethylacetamide (DMAc), methyl isobutyl ketone (MIBK), tetrahydrofuran (THF), and water (H_2_O). Among them, DMSO exhibited the highest catalytic efficiency for glucose conversion HMF.

**Figure 6:**
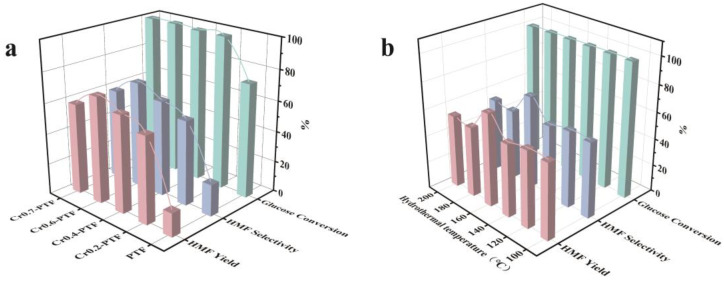
Effect of (**a**) Cr doping amount and (**b**) hydrothermal temperature on the catalytic activity of the prepared catalyst on catalytic activity under the following conditions: catalyst (0.075 g), glucose (0.15 g), NaClaq-DMSO biphasic system (NaClaq/DMSO: 2/18), 180 °C, 3 h.

**Figure 7:**
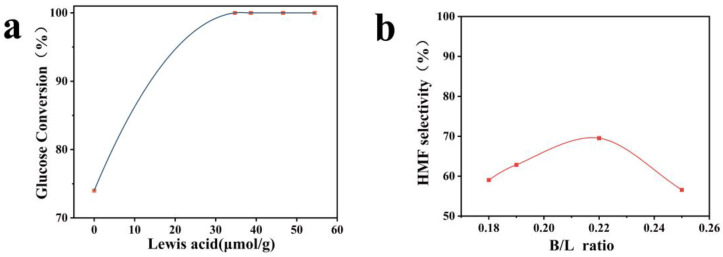
Effect of (**a**) Lewis acid on the glucose conversion and (**b**) B/L ratio on the selectivity to HMF under the following conditions: catalyst (0.075 g), glucose (0.15 g), NaClaq-DMSO biphasic system (NaClaq/DMSO: 2/18), 180 °C, and 3 h.

Figure 6a illustrates the catalytic performance of CrX-PTF in glucose dehydration to HMF. The chromium-loaded phenolic resin porous carbon catalyst outperformed both the unmodified phenolic resin porous carbon catalyst (PTF) and CrCl_3_·6H_2_O, which contains only Lewis acid sites. Under reaction conditions of 180 °C for 3 h, PTF achieved an HMF yield of 15.5% with 20.51% selectivity, while CrCl_3_·6H_2_O produced a 28.3% yield with 34.34% selectivity (Table S3). In contrast, Cr0.6-PTF demonstrated superior catalytic efficiency, reaching a maximum HMF yield of 69.5% under the same conditions. The HMF yields for Cr0.2-PTF, Cr0.4-PTF, and Cr0.7-PTF were 56.6%, 62.9%, and 59%, respectively (Table S3). Cr0.2-PTF exhibited the highest Brønsted/Lewis (B/L) acid ratio (0.25) but had lower total and Lewis acidity than Cr0.6-PTF (B/L = 0.22), leading to lower catalytic activity. Previous studies have shown that an optimal balance between Lewis and Brønsted acid sites is crucial for maximizing glucose conversion to HMF, rather than a higher B/L ratio necessarily improving HMF selectivity [44,47,48]. In this study, we analyzed the B/L ratio (Table 2, entry 5) and its correlation with HMF selectivity (Figure 7b). The highest HMF yield (69.5%) was obtained at an optimal B/L ratio of 0.22. When the B/L ratio decreased to 0.18 and 0.19, the HMF yield dropped to 59% and 62.9%, respectively, while increasing the ratio to 0.25 further reduced the yield to 56.6% (Table 2). Based on these findings, Cr0.6-PTF was identified as the optimal catalyst for further research on glucose-to-HMF conversion.

Figure 6b illustrates the impact of the hydrothermal temperature during catalyst preparation on the catalytic performance of the resultant catalyst. When the hydrothermal temperature was set to 100, 120, 140, 160, 180, and 200 °C, the HMF yields using Cr0.6-PTF as the catalyst were 56%, 57, 54%, 69.5%, 52%, and 54%, respectively, while the HMF selectivities were 56%, 57%, 54%, 69.5%, 52%, and 54%, respectively. Consequently, we selected a hydrothermal temperature of 160 °C as the optimal condition for catalyst preparation.

**Figure 8:**
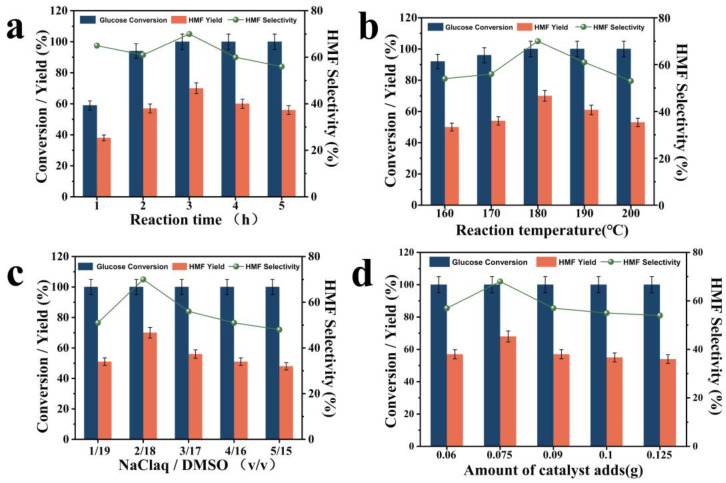
Effect of (**a**) reaction time (**b**) and temperature, (**c**) NaClaq/DMSO (*v*/*v*), (**d**) Cr0.6-PTF dosage on the catalytic activity under the following conditions: Cr0.6-PTF (0.075 g), glucose (0.15 g), NaClaq-DMSO biphasic system (NaClaq/DMSO: 2/18), 180 °C, and 3 h.

To determine the optimal conditions for HMF synthesis from glucose, the effects of reaction time, temperature, the ratio of NaClaq to DMSO, and catalyst dosage on the conversion of glucose to HMF using Cr0.6-PTF as a catalyst were examined. We also presented HPLC chromatograms for HMF detection under different reaction conditions (Figures S4 and S5). The results demonstrated that the reaction time and temperature significantly influenced HMF production from glucose. Figure 8a illustrates that the HMF yields were 37.8%, 57.8%, and 69.5% when the reaction time were 1, 2, and 3 h, respectively. However, further extending the reaction time to 4 and 5 h resulted in decreased HMF yields of 60.13% and 55.86%, respectively. The reaction temperature was varied from 160 to 200 °C (Figure 8b). The HMF yield increased from 50.1% to 69.5% and the HMF selectivity improved from 54% to 69.5% as the rection temperature was increased from 160 to 180 °C. However, increasing the reaction temperature beyond 180 °C enhanced glucose conversion while reducing HMF yield. Specifically, at reaction temperatures of 190 and 200 °C, the HMF yields were 61% and 53%, respectively, with corresponding HMF selectivities of 61% and 53%. An excessive reaction temperature promotes the formation of by-products, such as formic and acetylpropionic acids, substantially reducing the HMF yield [49,50]. Therefore, to mitigate the reduction in HMF yield, a reaction condition of 180 °C for 3 h was used in this experiment.

The NaClaq-to-DMSO ratio was optimized. The volume ratios of NaClaq to DMSO were set to 1/19, 2/18, 3/17, 4/16, and 5/15. The highest HMF yield (69.5%) was obtained at a ratio of 2/18 (Figure 8c). A lower ratio of NaClaq to DMSO (1/19) resulted in decreased HMF yields (51.5%), whereas higher ratios (3/17, 4/16, and 5/15) reduced yields (55.9%, 51.5%, and 48.5%, respectively). The influence of the NaClaq-to-DMSO ratio on HMF selectivity followed a similar trend, with selectivity values of 51.5%, 69.5%, 55.9%, 51.5%, and 48.5% for the respective ratios. Therefore, the optimal NaClaq/DMSO ratio was 2/18.

When the Cr0.6-PTF dosage was 0.06 g, the HMF yield was unsatisfactory at 57% (Figure 8d). Increasing the Cr0.6-PTF dosage to 0.075 g resulted in the highest HMF yield (69.5%). Using ICP-OES data (Table S5), the glucose-to-Cr molar ratios for catalyst dosages of 0.06, 0.075, 0.09, 0.1, and 0.125 g were calculated as 4.0, 3.2, 2.6, 2.4, and 1.9, respectively. These results show that higher catalyst loading increases the number of active acid sites, enhancing the reaction. However, excessive amounts of catalyst promote side reactions that consume HMF, as surplus acid sites facilitate glucose dehydration to fructose and HMF oligomerization [11]. Therefore, a Cr0.6-PTF dosage of 0.075 g was optimal and provided sufficient active sites. The findings substantiate that Cr0.6-PTF exhibits enhanced catalytic performance in glucose-to-HMF conversion compared to reported heterogeneous solid catalysts (Table 3).

**Figure 9:**
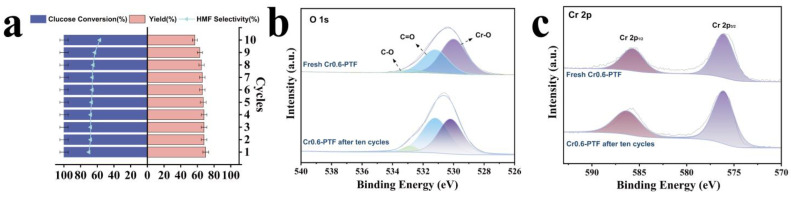
(**a**) Cr0.6-PTF recycling performance and (**b**) XPS O1s high-resolution and (**c**) Cr2p high-resolution spectra of Cr0.6-PTF after 10 cycles of use under the following conditions: Cr0.6-PTF (0.075 g), glucose (0.15 g), NaClaq-DMSO biphasic system (NaClaq/DMSO: 2/18), 180 °C, 3 h.

The recyclability of catalysts is of paramount importance for their potential industrial applications. To evaluate the reusability and stability of Cr0.6-PTF, 10 HMF production cycles were performed to evaluate its synthesis from glucose. After each cycle, Cr0.6-PTF was isolated from the product mixture via centrifugation, then rinsed with deionized water and dried in an oven at 70 °C for 8 h. Cr0.6-PTF exhibited sustained catalytic activity for the conversion of glucose to HMF in the ninth reaction cycle. The HMF yield was 69.5% during the initial reaction cycle. By the ninth cycle, the HMF yield had reached 63% (Figure 9a). However, during the 10th reaction cycle, the HMF yield decreased slightly to 57.5%. XPS analysis of the catalyst after 10 cycles revealed no significant changes in its bonding network compared to the fresh catalyst, confirming structural stability (Figure 9b,c). However, quantitative O1s analysis showed a 13.4% reduction in Cr-O coordination (from 63.3% to 49.9%), along with a 1.13% decrease in Cr content (from 5.11% to 3.98%) (Table S1), indicating a loss of reactive sites, likely contributing to the decline in catalytic performance. XRD spectra further supported the catalyst’s stability, as no significant structural changes were observed between the fresh and post-10-cycle catalysts (Figure S2).

The contribution of dissolved Cr ions to the glucose-to-HMF conversion was minimal, as shown in Figure S3b. After 3 h, dissolved Cr ions contributed only 0.17% to the HMF yield, demonstrating their negligible role in the catalytic process. Therefore, Cr0.6-PTF is the dominant catalyst in the heterogeneous reaction. Additionally, as shown in Figure S3a, the reaction solution was filtered at each time interval and the chromium concentration analyzed using atomic absorption spectroscopy (AAS). As indicated in Table S6, chromium leaching was minimal (only 0.8% of the total Cr content after 4 h), further confirming the catalyst’s stability. In summary, Cr0.6-PTF demonstrated excellent catalytic activity and reusability for glucose conversion to HMF in the NaClaq–DMSO biphasic system, outperforming other carbon-based catalysts (Table 4).

## 3. Materials and Methods

### 3.1. Materials

Ammonia solution (25–28wt%) and formaldehyde (37–40wt%) were purchased from Tianjin Zhiyuan Chemical Co., Ltd., Tianjin, China, and Pluronic F127 was purchased from Sigma-Aldrich, Ltd., St Louis, Waltham, MA, USA. HMF (99%), D-(+)-glucose, DMSO (99.8%), CrCl_3_·6H_2_O, TA, chromatography-grade methanol, and glacial acetic acid were purchased from Shanghai Aladdin Industrial Co., Ltd., Shanghai, China.

### 3.2. Catalyst Preparation

CrX-PTF catalysts were prepared with some modifications based on previously reported methods [32]. Briefly, 0.20 g of F127 was dissolved in 50 mL water. Next, 0.5 mL NH_3_·H_2_O was added. Following a 30 min interval, 0.2 g TA and 0.4 mL HCHO solution were introduced under magnetic stirring. The mixture was continuously stirred for 24 h before adding 0.1 g/mL CrCl_3_·6H_2_O solution. The CrCl_3_·6H_2_O-to-TA mass ratios for all chromium-doped samples are presented in Table 5. The Cr-doped phenolic resin composites were prepared via a hydrothermal process at 100, 120, 140, 160, 180, and 200 °C for 4 h, followed by centrifugation, washing, and drying. The resulting samples were labeled CrX-PTF, where X indicated the quantity of added metal. When the TA/CrCl_3_·6H_2_O mass ratios were 1:1, 1:2, 1:3, and 1:3.5, the corresponding X values were 0.2, 0.4, 0.6, and 0.7, respectively. The material synthesized without adding metal solution was designated as PTF.

### 3.3. Catalytic Reaction

The catalytic reaction was conducted in a high-temperature oven using a 100 mL Teflon-lined autoclave. Briefly, 0.15 g of glucose, the catalyst, and 20 mL of solvent (saturated NaCl solution and DMSO) were added to an autoclave and sealed. The autoclave was heated to the desired temperature (160, 170, 180, 190, or 200 °C), and the temperature was maintained for 1, 2, 3, 4, or 5 h, respectively. Upon completion of the reaction, the reactor was rapidly cooled to 20–35 °C in an ice water bath. The resulting mixture was then subjected to centrifugation at a speed of 9000 r/min for 5 min. The liquid phase was analyzed using high-performance liquid chromatography (HPLC). For the stability tests, the solid catalyst was washed multiple times with ethanol and deionized water, followed by drying for use in subsequent cycles.

### 3.4. Characterization

The detailed characterization properties are presented in the Supporting Information (Text S1).

### 3.5. Product Analysis

The HMF and glucose concentrations were analyzed using HPLC. A ZORBAX Eclipse XDC-C18 column (Element Lab Solutions, Scotland, UK) at 30 °C with ultraviolet detection at 285 nm was used for HMF analysis. The mobile phase consisted of 1% acetic acid and methanol (9: 1, *v*/*v*) at a 1 mL/min flow rate. A Bio-Rad Aminex HPX-87H column (Bio-Rad Laboratories, Hercules, CA, USA) at 50 °C with refractive index detection and 5 mmol/L H_2_SO_4_ as the mobile phase at 0.60 mL/min was used for glucose analysis. Both analyses used an injection volume of 20 μL of the 1filtered solution. The glucose conversion, HMF yield, and HMF selectivity were calculated using the following formulae:

(1) $${\mathrm{Glucose}}_{\mathrm{conversion}}=(1-\frac{\mathrm{moles}\;\mathrm{of}\;\mathrm{remaining}\;\mathrm{glucose}}{\mathrm{moles}\;\mathrm{of}\;\mathrm{staring}\;\mathrm{stach}})\times 100\%$$

(2) $${\mathrm{HMF}}_{\mathrm{yield}}=\frac{\mathrm{moles}\;\mathrm{of}\;\mathrm{HMF}\;\mathrm{in}\;\mathrm{solution}}{\mathrm{moles}\;\mathrm{of}\;\mathrm{starch}\;\mathrm{in}\;\mathrm{the}\;\mathrm{reaction}}\times 100\%$$

(3) $${\mathrm{HMF}}_{\mathrm{selectivity}}=(\frac{{\mathrm{HMF}}_{\mathrm{yield}}}{{\mathrm{Glucose}}_{\mathrm{conversion}}})\times 100\%$$

## 4. Conclusions

In this study, a Cr-doped phenolic resin porous hydrothermal carbon catalyst was successfully synthesized to convert glucose to HMF. Owing to the coexistence of Cr_2_O_3_ NPs and phenolic-resin-based hydrothermal carbon within the catalyst, CrX-PTF exhibited bifunctional heterogeneous catalytic properties, possessing Bronsted and Lewis acid sites. The Cr0.6-PTF catalyst was the most active, achieving a high HMF yield of 69.5% at 180 °C for 3 h in a NaClaq-DMSO biphasic system. As a novel catalyst, Cr0.6-PTF exhibited considerable potential for industrial applications because of its environmentally friendly preparation process, exceptional activity, and superior reusability. This study presents a practical approach for the design of supported metal–phenolic-resin-based bifunctional heterogeneous catalysts.

## Figures and Tables

**Table 1:** Pore parameters of different catalysts.

Sample	S_BET_ (m^2^/g)	Total Pore Volume (cm^3^/g)	Micropore Volume (cm^3^/g)	Mesopore Volume (cm^3^/g)	Pore Size (nm)
Cr0.2-PTF	126.54	0.57	0.0013	0.57	18.09
Cr0.4-PTF	97.31	0.21	0.0024	0.21	8.47
Cr0.6-PTF	39.75	0.06	0	0.06	6.14
Cr0.7-PTF	33.63	0.05	0	0.05	4.11
PTF	21.54	0.12	0	0.12	17.61

**Table 2:** Brønsted/Lewis and total acids in various catalysts determined using pyrolysis–Fourier transform infrared spectroscopy.

Sample	Brønsted Acidity (μmol/g)	Lewis Acidity (μmol/g)	Total Acidity (μmol/g)	B/L Ratio
PTF	10.54	0	10.54	
Cr0.2-PTF	8.64	34.73	43.37	0.25
Cr0.4-PTF	7.38	38.68	46.06	0.19
Cr0.6-PTF	10.37	46.62	56.99	0.22
Cr0.7-PTF	9.83	54.43	64.25	0.18

PTF—polyphenol-formaldehyde resin, B/L ratio—Brønsted/Lewis acid ratio.

**Table 3:** Overview of HMF yields from glucose for different catalysts under optimal conditions.

Catalyst	Solvent	T (°C)	Reaction Time (h)	HMF Yield (%)	HMF Selectivity (%)	References
Cr0.6-PTF	NaClaq/DMSO	180	3	69.5	69.5	This work
LS-Cr	NaClaq/THF	190	4	60.4	60.4	[51]
GO-Cr_2_O_3_	[EMIM]Br	140	4	67	-	[52]
FPIL2a	H_2_O/DMSO	150	2	48.7	-	[53]
Cr-HAP	[EMIM]Br	150	0.5	32.5	37.7	[54]
Cr(salen)-IM-HSO4-MCM-41	DMSO	140	4	43.5	43.5	[55]
Cr(Salten)-MCM-41-[(CH_2_)-_3_SO_3_HVIm]HSO_4_	DMSO	140	4	50.2	50.2	[56]

**Table 4:** Evaluation of the catalytic efficiency for converting glucose to HMF over multiple cycles.

Catalyst	Solvent	T (°C)	Reaction Time (h)	HMF Yield Using Recycled Catalyst (%)	Catalyst Recycling Runs	References
Cr0.6-PTF	NaClaq/DMSO	180	3	62	10	This work
Zn-modified activated carbon	NaClaq/THF	160	8	51	3	[57]
Sn-Mont	THF/DMSO	160	3	53.5	4	[58]
CnS700-Sn	A-NADES	140	2	22.3	6	[59]
SiO_2_-Zr-P	NaClaq/THF	180	3	69	5	[60]
0.4-Cr/β zeolite	NaClaq/THF	150	1.5	58	3	[61]

**Table 5:** Chemicals added to different catalyst samples.

Catalyst	NH_3_·H_2_O (mL)	HCHO (mL)	TA (g)	CrCl_3_·6H_2_O (g)	TA/CrCl_3_·6H_2_O
PTF	0.5	0.4	0.2	0	
Cr0.2-PTF	0.5	0.4	0.2	0.2	1:1
Cr0.4-PTF	0.5	0.4	0.2	0.4	1:2
Cr0.6-PTF	0.5	0.4	0.2	0.6	1:3
Cr0.7-PTF	0.5	0.4	0.2	0.7	1:3.5

## Data Availability

The data supporting the findings of this study are available from the corresponding author upon request.

## Supplementary Materials

The following supporting information can be downloaded at: https://www.mdpi.com/article/10.3390/ijms26083648/s1.
