# Supplementary material for: Bifunctional Chromium-Doped Phenolic Porous Hydrothermal Carbon Catalysts for the Catalytic Conversion of Glucose to 5-Hydroxymethylfurfural
Source: Int J Mol Sci. 2025 Apr 12;26(8):3648. doi: 10.3390/ijms26083648 (PMC12027228; doi:10.3390/ijms26083648)
Supplement: Supplementary file 1 [file ijms-26-03648-s001.zip › ijms-3547197-supplementary.pdf]

Bifunctional chromium-doped phenolic porous hydrothermal carbon catalysts for the catalytic conversion of glucose to 5-hydroxymethylfurfural

Pize Xiao [a], Wei Mao [a], Zhiming Wu [a], Huimin Gao [a], Chutong Ling [a], Jinghong Zhou [a*]

[a] Guangxi Key Laboratory of Clean Pulp & Papermaking and Pollution Control, School of Light Industrial and Food Engineering, Guangxi University, Nanning 530004, China

* Corresponding author, E-mail: jhzhoudou@gxu.edu.cn

No. 100, East University Road, Nanning, Guangxi 530004, China.

Text S1 Characterization

A powder X-ray diffractometer (XRD [Rigaku MiniFlex600, Rigaku, Tokyo, Japan]) with Cu Kα radiation was used to characterize the crystalline phases of the catalysts, scanning from 10 to 90° 2θ at 10°/min. A nitrogen adsorption-desorption analyzer (Micromeritics ASAP 2460 [Micromeritics Instrument Corporation, Norcross, USA]) was used to measure the specific surface area, total pore volume, and pore size distribution. The samples were degassed at 120 °C under vacuum for 8 h before analysis, and the surface area and pore volume were calculated using the Brunauer-Emmett-Teller (BET)) and Barrett-Joyner-Halenda methods. Thermal gravimetric analysis was performed on an EXSTAR 7200 thermo-analyzer (EXSTAR, Co., Ltd., Tokyo, Japan) from 30 to 800 °C at 10 °C/min. Scanning (SEM, ZEISS Sigma 300, Carl Zeiss, Oberkochen, Germany) and transmission electron microscopes (TEM, JEOL JEM-F200, JEOL Ltd., Tokyo, Japan) were used to determine the morphology and surface composition. Brønsted and Lewis acid sites were analyzed using pyridine-adsorbed Fourier Transform Infrared (Py-FTIR) spectroscopy using a Bruker Tensor 27 instrument (Bruker Optik, Ettlingen, Germany). An X-ray photoelectron spectroscopy (XPS) was used to determine the elemental valence states. Inductively coupled plasma optical emission spectroscopy (ICP-OES) was employed to determine the metal element content in the catalysts, while atomic absorption spectroscopy (AAS) was utilized to measure metal leaching amounts.

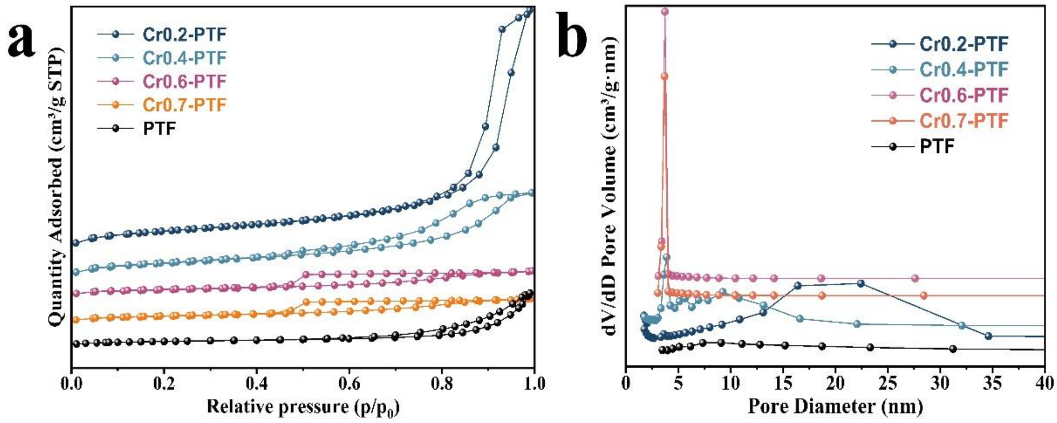

Figure S1. (a) N_2 isotherms and (b) pore size distributions of different catalysts.

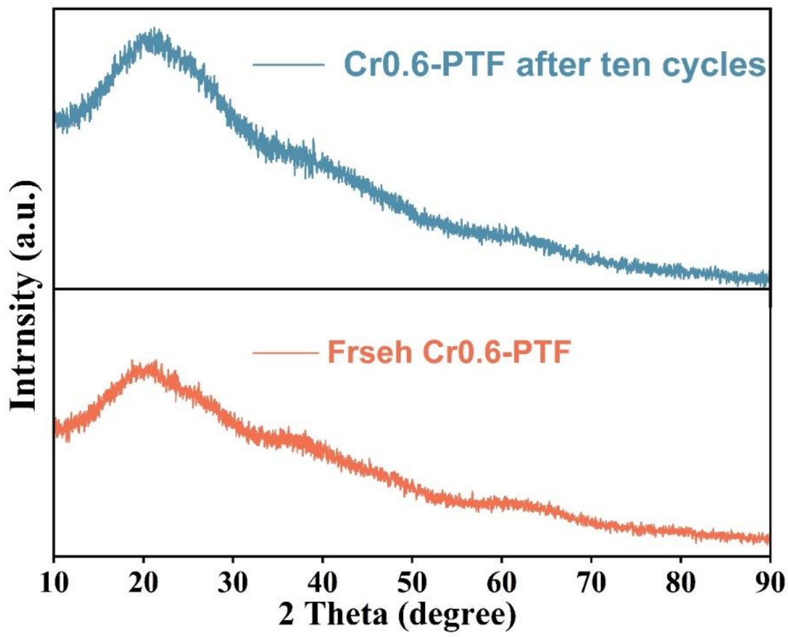

Figure S2. Cr0.6-PTF XRD patterns before and after 10 cycles

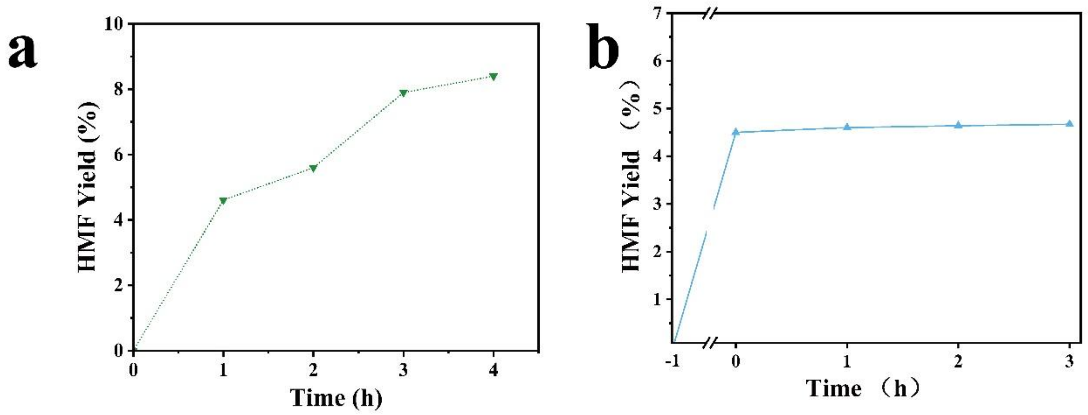

Figure S3. Cr0.6-PTF catalyst hot filtration experiment. (a) HMF yield with Cr0.6-PTF continuously present in the reaction; (b) HMF yield from the reaction using the filtrate after the Cr0.6-PTF catalyst was removed by hot filtration following 1hour of reaction. Reaction conditions: 100°C, 0.15 g glucose, 0.075 g catalyst, 18mLDMSO, 2mL NaClaq.

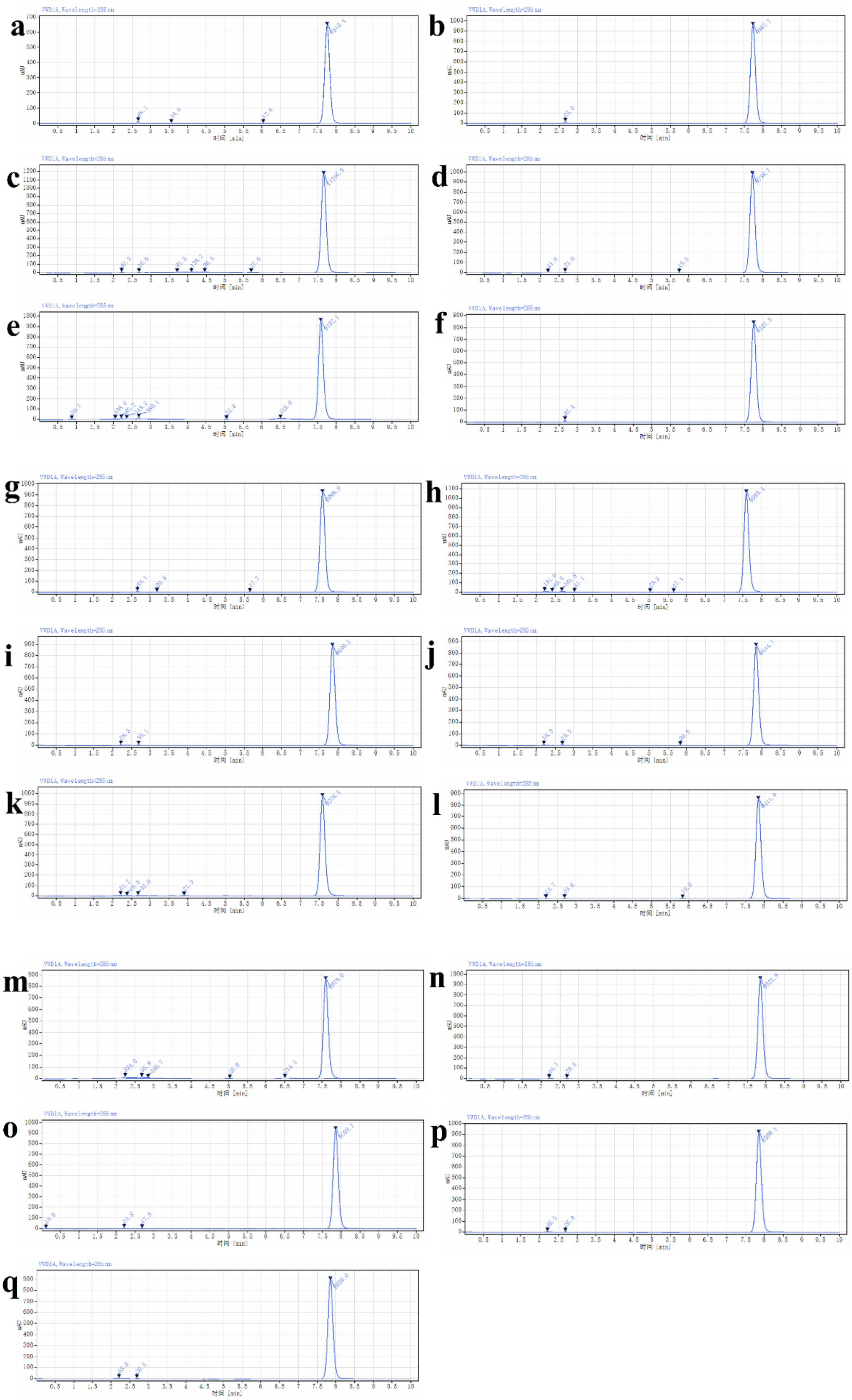

Fig.S4. HPLC chromatograms of various samples. (a-e): different reaction times; (f-i): different reaction temperatures; (j-m): Varying NaCl(aq)/DMSO ratios; (n-q): different Cr0.6-PTF dosage.

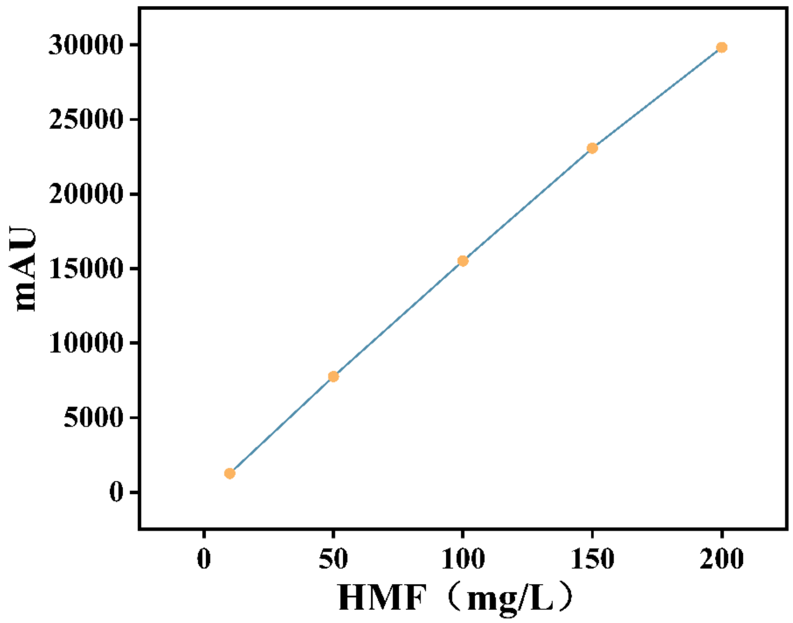

Figure S5. HPLC standard curve for HMF, with the equation: y = 150.85x + 103.66, and a linear correlation coefficient R^2 = 0.999.

Table S1. Surface composition of catalysts by XPS

Catalyst	Content(at.%)			C(%)				O（%）
	C	O	Cr	C-H	C-C	C-O	C=O	Cr-O
PTF	75.66	24.34	0.0	48.39	38.17	12.04	1.4	0
Cr0.2-PTF	59.35	34.88	1.16	40.86	39.23	13.34	6.57	53.98
Cr0.6-PTF	57.15	37.74	5.11	52.29	32.72	7.02	7.97	63.30
Cr0.6-PTF after 10 cycles	65.23	30.79	3.98	70.1	15.3	8.1	6.5	49.9

Table S2. Residual CrX-PTF content from thermogravimetric analysis (TGA).

Sample	Cr-PTF content (wt.%)
Cr0.2-PTF	18.88
Cr0.3-PTF	23.06
Cr0.4-PTF	26.41
Cr0.5-PTF	29.56
Cr0.6-PTF	30.67

Table S3. Glucose Conversion to HMF using different catalysts.

Catalyst	HMF yield (%)	HMF selectivities (%)	Glucose conversion (%)
PTF	15.15	20.51	74
$CrCl_3 \cdot 6H_2O$	28.30	34.34	82
Cr0.2-PTF	56.57	56.57	100
Cr0.3-PTF	58.44	58.44	100
Cr0.4-PTF	62.85	62.85	100
Cr0.5-PTF	65.71	65.71	100
Cr0.6-PTF	69.53	69.53	100
Cr0.7-PTF	59.04	59.04	100

Reaction conditions: 0.15 g glucose, 0.075 g catalyst, 18mLDMSO, 2mL NaClaq.

Table S4. Effect of solvent type on HMF yield and selectivity

Catalyst	HMF yield (%)	HMF selectivities (%)	Glucose conversion (%)
DMSO/$NaCl_{aq}$	69.5	69.5	100
MIBK/$NaCl_{aq}$	46.9	46.9	100
GVL/$NaCl_{aq}$	50.1	50.1	100
DMF/$NaCl_{aq}$	19.4	19.4	100
DMAc/$NaCl_{aq}$	15.8	15.8	100
THF/$NaCl_{aq}$	46.1	46.1	100
H_2O	16.7	16.7	100

Reaction conditions：Cr0.6-PTF (0.075 g), glucose (0.15 g), NaClaq- solvent biphasic system (V/V: 2/18), 180 ℃, 3 h.

Table S5. Cr Loading in Catalysts Determined by ICP-OES Analysis

Catalyst	Cr loading in catalyst (%)
Cr0.2-PTF	10.8
Cr0.3-PTF	12.6
Cr0.4-PTF	15.6
Cr0.5-PTF	17.2
Cr0.6-PTF	18.2

Table S6. Chromium Leaching from the Cr0.6-PTF Catalyst

Time (h)	Leaching Percentage of Cr (%)
1	0.2
2	0.5
3	0.6
4	0.8
